# Effectiveness of negative pressure wound therapy in Ludwig’s angina: a retrospective study of 18 cases

**DOI:** 10.1186/s12893-025-02957-y

**Published:** 2025-05-22

**Authors:** Shu-Jun Chen, Ning Ji, Yu-Xuan Chen, Jian-Rui Xiao, Xiao-Zong Wei, Yan-Kun Liu

**Affiliations:** 1 Department of Stomatology, the 82nd Group Army Hospital of PLA, No. 991, Baihuadong Road, Baoding, 071000 P. R. China; 2 Quality Management Department, the 82nd Group Army Hospital of PLA, No. 991, Baihuadong Road, Baoding, P. R. China 071000

**Keywords:** Negative pressure wound therapy, Ludwig’s angina, Necrotizing soft tissue infection, Necrotizing fasciitis

## Abstract

**Objective:**

To investigate the effectiveness of negative pressure wound therapy (NPWT) for Ludwig’s angina (LA).

**Methods:**

We retrospectively reviewed 18 patients with LA admitted to the 82nd Group Army Hospital of PLA between October 2014 and October 2021. All patients underwent surgical drainage and debridement within 6 h after admission. A minimally invasive approach involving bilateral small incisions in the submandibular area was used to perform the procedure. An NPWT device was applied for positive drainage of the involved spaces after debridement. Postoperatively, the patients received the appropriate supportive care and antibiotic therapy. Data collection encompassed sex, age, systemic diseases, dressing change frequency, NPWT duration, wound healing time, and ICU stay length. Follow-up was performed to evaluate recurrence, scarring, and neck mobility. For comparative analysis, control data were obtained from LA patients treated with conventional surgical drainage between January 2008 and September 2014. Descriptive statistics and Student’s t-test were employed for statistical analysis.

**Results:**

In the NPWT group, all patients had uneventful courses during hospitalization and were discharged upon complete wound healing. Fifteen patients required only a single session of surgical debridement with NPWT, while the remaining three underwent two procedures. Upon NPWT device removal, all infectious cavities exhibited clean wounds with mature granulation tissue formation. Compared to the conventional surgery group, the NPWT group demonstrated a significantly shorter wound healing time (15.33 ± 3.93 vs. 19.50 ± 2.17 days; *p* = 0.025), reduced ICU stay duration (0.61 ± 0.61 vs. 2.17 ± 0.75 days; *p* < 0.001) and markedly fewer dressing changes (2.17 ± 0.38 vs. 17.00 ± 3.16; *p* < 0.001).

**Conclusions:**

NPWT demonstrated excellent effectiveness in the management of LA. Compared to conventional surgical debridement and drainage, it offers several distinct clinical advantages, including accelerated wound healing, shortened ICU stays, and reduced dressing change frequency. These benefits are clinically linked to both reduced postoperative pain perception and decreased nursing workload. Additionally, smaller incisions result in less surgical trauma and improved cosmetic outcomes. NPWT should be considered as a viable approach in the management of LA. Future randomized controlled trials are needed to confirm NPWT’s superiority in larger cohorts.

**Clinical trial number:**

Not applicable.

## Introduction

Ludwig’s angina (LA), first described by the German physician Wilhelm Friedrich von Ludwig in 1836, is a severe soft tissue infection involving the submental space, bilateral sublingual spaces, and submandibular spaces. LA progresses rapidly; if not treated promptly, it can lead to serious complications such as respiratory obstruction and sepsis. Further spread of infection to the lower neck may extend to the mediastinum, resulting in descending necrotizing mediastinitis (DNM), a highly fatal complication [1–4]. The causative organisms of LA are typically *Streptococcus* species or mixed pathogens combined with anaerobes. The key to improving cure rates lies in early empirical administration of broad-spectrum antibiotics and aggressive surgical debridement. The classic surgical approach involves large transverse incisions connecting both submandibular regions, or even inverted T-shaped incisions, which often cause significant surgical trauma and postoperative scarring issues [5]. Moreover, frequent postoperative dressing changes (ranging from single to multiple daily sessions) and necrotic tissue removal impose substantial pain on patients and a considerable workload burden on healthcare staff.

Negative pressure wound therapy (NPWT) is a therapeutic method using a special sponge or gauze dressing with a vacuum device to promote wound healing. It has proven highly effective in wound management, with its primary mechanisms focusing on mechanically reducing local exudate, enhancing tissue perfusion, and promoting granulation tissue formation. NPWT, commonly referred to as vacuum-assisted closure (VAC) in the English literature, has been widely applied to treat acute and chronic wounds in areas such as the chest, abdomen, perineum, and extremities [6, 7]. Furthermore, NPWT has gained broad clinical acceptance in managing complex wound infections. Existing studies have demonstrated its clear advantages over conventional wound care in terms of reduced healing time, shorter hospital stays, and decreased duration of antibiotic therapy [8].

In head and neck surgery, NPWT is commonly employed for wound repair; however, its application in facial and neck infections has been primarily restricted to wound closure after infection control [9, 10]. Most existing reports on such cases are limited to single case studies or small case series. Recent studies have demonstrated favorable outcomes with NPWT in managing head and facial infections [11, 12]. Nevertheless, the use of NPWT specifically for LA remains insufficiently explored. Given NPWT’s established efficacy in controlling various types of wound infections, we believe it can offer a novel therapeutic approach for LA.

We have been using NPWT for the treatment of LA since 2014 and the clinical efficacy of this system has been investigated.

## Materials and methods

This study was approved by the Ethics Committee of the 82nd Group Army Hospital of PLA. From October 2014 to October 2021, a total of 18 patients with LA were admitted to the 82nd Group Army Hospital of PLA (formerly the 252nd hospital of the People’s Liberation Army), and all cases were confirmed by physical examination, imaging tests and surgical operation. The inclusion criteria: (1) The infection involved the submental space, bilateral submandibular space and sublingual space. (2) Necrotizing infection was confirmed by surgical exploration including necrosis of fascia and positive “finger separation”.

All patients were evaluated regarding their history of hormone use, systemic medical conditions, dental history, and antibiotic usage following disease onset. Upon admission, routine laboratory investigations were conducted, including complete blood count, C-reactive protein levels, electrolyte analysis, liver and kidney function tests, and CT imaging. A comprehensive physical examination with dental assessment was also performed. All patients received intravenous imipenem or meropenem therapy. Surgical debridement was completed within 6 h of admission.

**Fig. 1:**
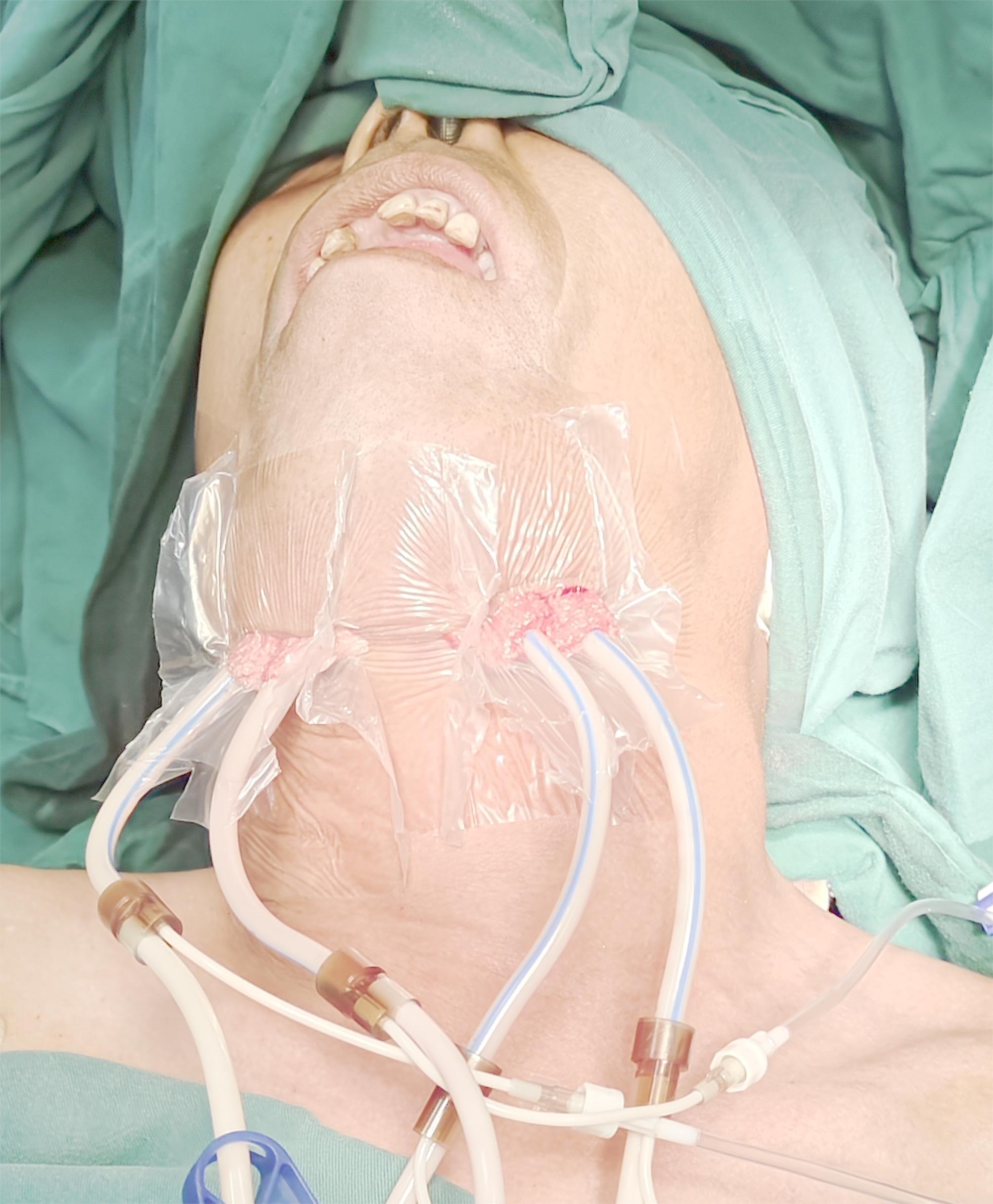
Drainage was performed with a suitable NPWT material according to the shape of the cavity, and the incisions were sealed with a semipermeable membrane

The operation was carried out under general anesthesia, before that a pus sample was collected for bacterial culture involving anaerobic and ordinary analysis via puncture. Surgical incisions approximately 3–4 cm in length were made bilaterally in the submandibular regions anterior to the mandibular angles, 2 cm below the lower border of the mandible. The skin, subcutaneous tissue, and platysma muscle were sequentially incised. Blunt dissection with the finger was performed within the submandibular space, extending anteriorly to the submental space to communicate with the contralateral submandibular space, and superiorly through the posterior border of the mylohyoid muscle to access the sublingual space. After removal of the necrotic tissue, a large amount of 3% hydrogen peroxide and 0.9% sodium chloride solution were used to flush the cavities. An NPWT device (Wuhan VSD Medical Science & Technology Co., Ltd., Wuhan, China) was applied for active drainage of the involved spaces according to the following protocol: (1) the peri wound skin was cleansed and dried; (2) VSD sponge material was trimmed to match the cavity morphology; (3) the material was secured with a semi-permeable membrane; and (4) seal integrity was verified (Fig. 1). Postoperative endotracheal intubation decisions were guided by clinical assessment of cervical swelling severity and respiratory status. The NPWT system was connected to a centralized vacuum source maintaining continuous negative pressure at -125 mmHg. Catheter patency was maintained through twice-daily saline irrigation. Systemic inflammatory response was monitored via serial measurements of complete blood count, C-reactive protein levels, and body temperature. Repeat CT imaging was obtained on postoperative day 3 to assess infection control status and NPWT device positioning adequacy. NPWT dressings were either removed or replaced 5–7 days postoperatively based on infection progression. For cases achieving adequate infection control, secondary closure with closed suction drain placement was performed, with drains retained for 48 h post-closure. Drains were removed until the wound closed. Patients underwent 1.5-2 years of follow-up post-discharge, during which submandibular scar appearance and cervical range of motion were evaluated.

As a historical control, data were collected on 6 patients diagnosed with LA between January 2008 and September 2014, who received traditional surgical drainage. Traditional broad neck incisions, connecting the submental and bilateral submandibular areas, were performed. After debridement, rubber drainage tubes with several side holes were placed in the involved cavities. Initially, the dressing was changed twice a day and the pus cavities were rinsed with hydrogen peroxide and normal saline each time to clean up the large amount of pus. The frequency of dressing changes was reduced according to the clinical situation. The incisions were sutured when the wound had developed fresh granulation tissue with no obvious purulent exudate.

The quantitative data were processed and analyzed using an online data analysis platform, SPSSPRO (https://www.spsspro.com). For group comparisons, the independent samples *t*-test was applied when the assumption of homogeneity of variance was met; otherwise, Welch’s *t*-test (WT) was utilized for statistical analysis.

## Results

The demographic and clinical characteristics of the patients were summarized in Table 1.

**Table 1:** The demographic and clinical characteristics of the patients

Variable	NPWT group (n = 18)	Traditional group (n = 6)
Mean age (years)	53.37	51.83
Range	35–82	18–76
Sex		
Male	11 (61.11%)	4 (66.67%)
Female	7 (38.89)	2 (33.33%)
Systemic disease		
Diabetes mellitus	7 (38.89%)	2 (33.33%)
Hypertension	8 (44.44%)	3 (50.00%)
Airway obstruction	10 (55.56%)	3 (50.00%)
NPWT frequency		
Once Twice	15 (83.33%) 3 (16.67%)	

In NPWT group, there were 11 males and 7 females with an average age of 53.37 years, ranging from 35 to 82 years. The most common cause was odontogenic infection in 17 patients, all of whom had posterior mandibular periodontitis or apical periodontitis, and in one patient the source was unknown. None of the patients had a history of NSAID use, and 12 patients had a history of systemic diseases, including 5 with diabetes, 8 with hypertension, 2 with rheumatoid disease, and 1 with asthma. Twelve patients had a history of toothache before disease onset. All patients used antibiotics after disease onset, including self-oral antibiotics in 4 patients and intravenous antibiotics in 14 patients.

**Fig. 2:**
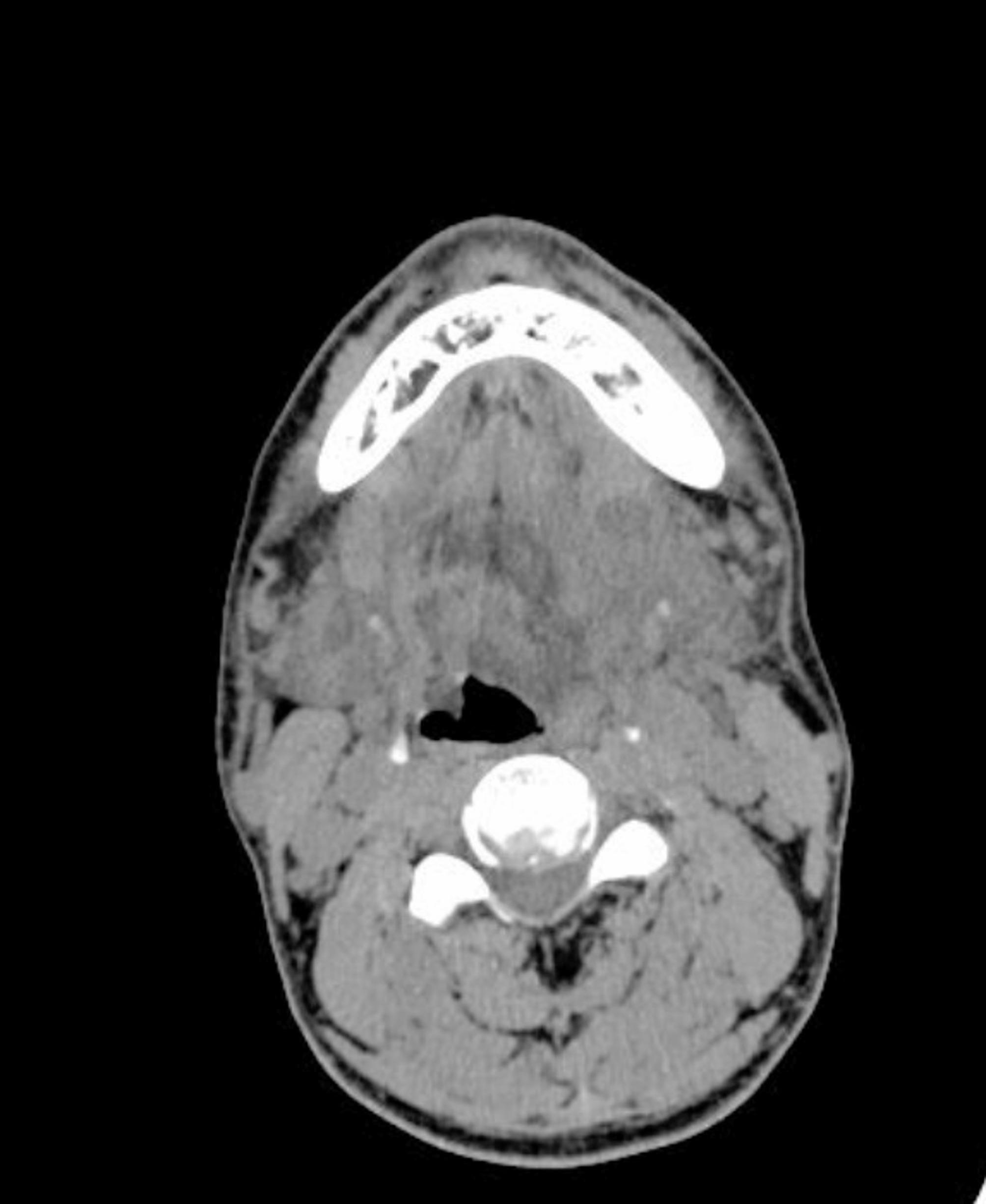
Axial CT at the level of the neck revealed diffuse swelling in the floor of the mouth and the submandibular regions

**Fig. 3:**
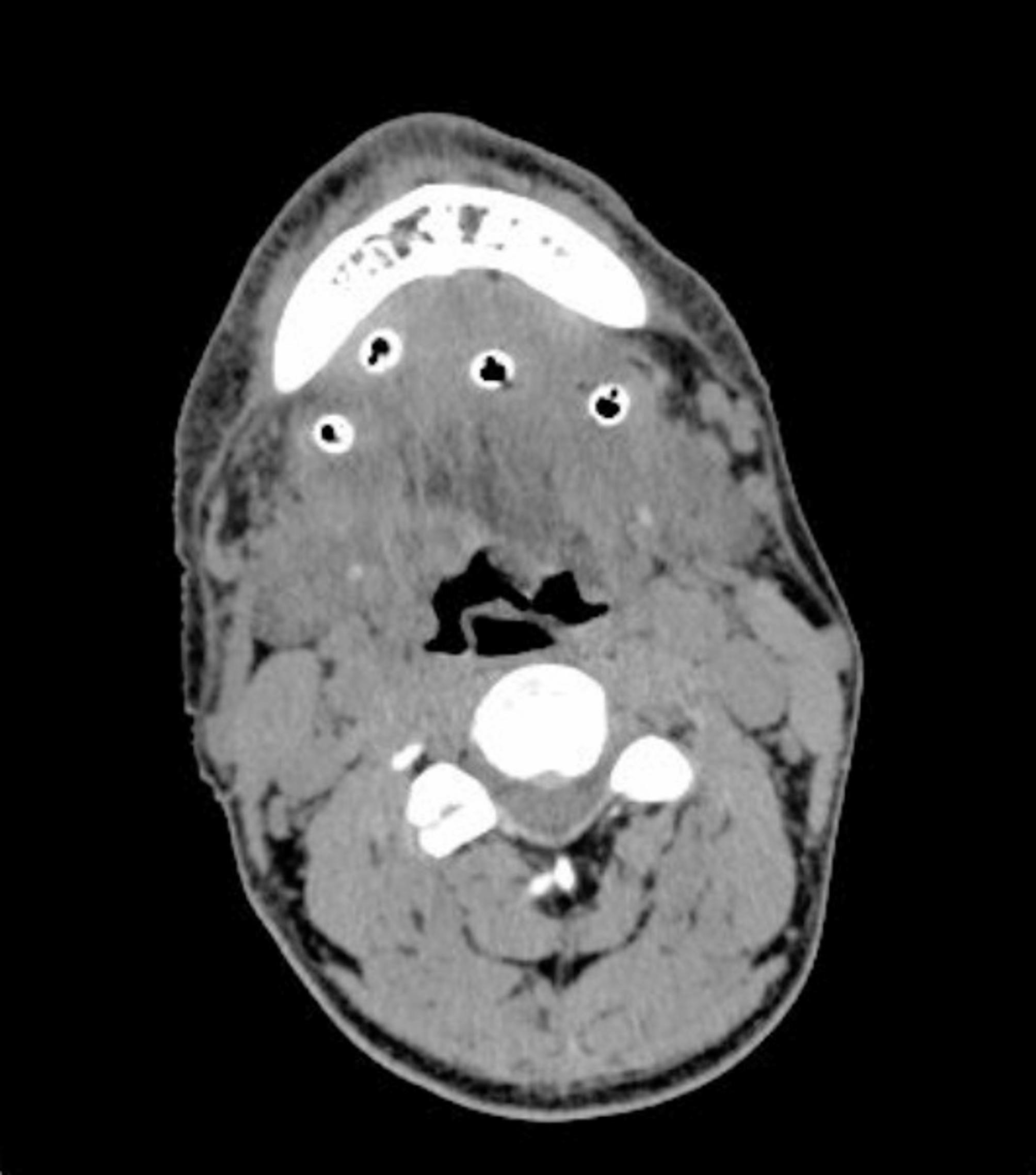
A postoperative axial CT image showed proper NPWT device placement

**Fig. 4:**
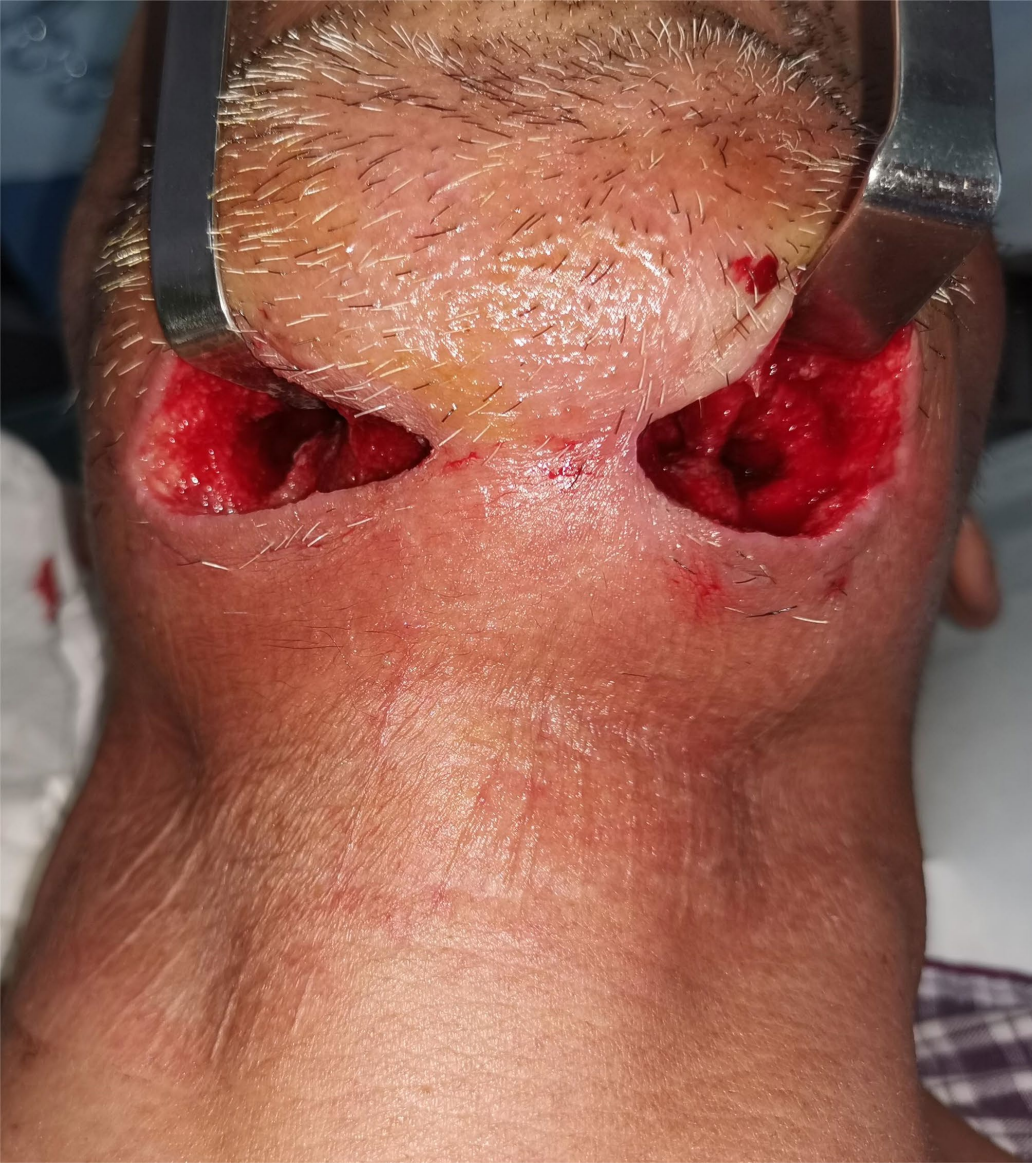
The infectious spaces showed a clean wound with healthy granulation formation during removal of the NPWT device

The median white blood cell count was 16.19 × 10^9^/L, with a range from 7.70 to 28.26 × 10^9^/L. The concentration of C-reactive protein ranged from 3.2 to 23.1 mg/dL. Among the 18 patients, 5 exhibited elevated aminotransferase levels, the most common hepatic abnormality. Sixteen patients presented with electrolyte disturbances, predominantly hypocalcemia (*n* = 13), followed by hyponatremia and hypochloremia (*n* = 12 each). CT scans of the neck revealed diffuse swelling in the floor of the mouth and the submandibular region (Fig. 2), with gas formation in 11 patients (61.11%). Ten patients manifested airway compromise at admission, including one with orthopnea. Postoperatively, 8 patients achieved successful extubation and transitioned to general ward care. The remaining 10 patients required intensive care unit (ICU) admission, with nasotracheal intubation maintained for 6–24 h (*n* = 9) or 48 h (*n* = 1). Repeated CT examinations confirmed optimal drainage device positioning (Fig. 3). Fifteen patients underwent single-stage surgical debridement with NPWT. Upon NPWT removal, all infected cavities displayed robust granulation tissue without purulent discharge (Fig. 4). Three patients required NPWT reapplication due to residual necrotic tissue identified during initial device removal, after which treatment progressed without complications. No NPWT-associated adverse events—including cutaneous ischemia, hemorrhage, or device malfunction—were documented.

A comparison of mean healing time, ICU stay duration, and dressing change frequency between the two groups is summarized in Table 2. The NPWT group exhibited significantly shorter mean wound healing time (15.33 ± 3.93 days vs. 19.50 ± 2.17 days; *P* = 0.025, independent samples *t*-test) and reduced ICU stay duration (0.61 ± 0.61 days vs. 2.17 ± 0.75 days; *P* < 0.001, independent samples *t*-test) compared to the conventional treatment group. Additionally, the NPWT group required markedly fewer dressing changes (2.17 ± 0.38 vs. 17.00 ± 3.16; *P* < 0.001, Welch’s *t*-test).

**Table 2:** Comparison of treatment of two groups

Variable	NPWT group (n = 18)	Traditional group (n = 6)	T value	*P* value
Wound healing time	15.33 ± 3.93	19.50 ± 2.17	-2.404	P =.025
ICU stay	0.61 ± 0.61	2.17 ± 0.75	-5.128	P <.001*
Dressing change frequency	2.17 ± 0.38	17.00 ± 3.16	-11.462	P <.001*

All 18 patients recovered and were discharged, and there were no in-hospital mortalities. During a follow-up period of 1.5 − 2 years with NPWT, 3 patients were lost to follow-up, one patient died of other diseases, and the remaining 14 patients were evaluable. Among these, 10 had clinic visits according to the follow-up schedule. 10 patients maintained regular clinical surveillance, demonstrating optimal therapeutic outcomes: (1) Well-epithelialized submandibular scars without hypertrophic formation; (2) Preserved cervical mobility. The follow-up data of the patients with traditional treatment were not available.

## Discussion

Ludwig’s angina (LA), a rapidly progressive necrotizing soft tissue infection of the submandibular and sublingual spaces, remains a rare clinical entity. Current literature predominantly describes LA within the broader category of deep neck infections rather than recognizing it as a distinct clinicopathologic syndrome. Pathologically, LA is characterized by fascial plane necrosis extending through the sublingual, submental, and submandibular compartments, often classified as necrotizing fasciitis of the oral floor. Standard management involves early broad-spectrum antibiotics (including anaerobic coverage), radical surgical debridement, and intensive supportive care [13, 14]. Notably, anatomically confined LA presentations are uncommon; most deep neck infections exhibit unilateral submandibular space involvement with variable submental extension. In contrast, LA frequently demonstrates rapid bilateral spread to parapharyngeal and cervical spaces. This distinct progression pattern complicates direct comparisons with other deep neck infections, necessitating strict diagnostic criteria for cohort homogeneity. Our study exclusively enrolled patients meeting definitive LA diagnostic standards. Despite therapeutic advances reducing mortality from > 50% to < 10%, LA persists as a high-acuity condition in oral and maxillofacial surgery. Treatment delays risk catastrophic sequelae including DNM, airway obstruction, septic shock, and multiorgan failure [13–15]. DNM itself carries a 17.5% mortality rate despite modern critical care, often requiring complex reconstruction with vascularized tissue flaps [4, 16].

As a necrotizing cervical infection, LA typically involves polymicrobial pathogens including both aerobic and anaerobic organisms [17–19]. In our series, microbiological cultures identified only two pathogens: *Klebsiella pneumoniae* and *Staphylococcus aureus*. This low culture positivity rate likely reflects prior antibiotic administration before admission, though such pretreatment does not compromise diagnostic certainty or therapeutic efficacy [20]. Notably, 11 of 18 patients exhibited gas-containing abscesses within involved spaces on CT imaging—a radiographic hallmark of necrotizing fasciitis and gas-forming bacterial activity.

Antimicrobial therapy remains pivotal in LA management. Current guidelines recommend combining β-lactams with nitroimidazoles to target gram-negative bacilli and anaerobes, while carbapenems are reserved for severe cases requiring broad-spectrum coverage. In our series, intravenous meropenem or imipenem achieved rapid infection control without drug-related adverse events.

Surgical treatment is considered key to reducing mortality [21, 22]. Traditional approaches employ extensive cervical incisions connecting submental and bilateral submandibular regions, occasionally extending to inverted T-shaped configurations to ensure complete drainage—a strategy associated with substantial iatrogenic trauma. Postoperative care complexities are compounded by frequent dressing changes (2–4 times daily) and repeated debridement, imposing significant pain burdens on patients. Clinically, each dressing change requires two healthcare personnel and approximately 30 min to complete, representing considerable resource utilization.

NPWT addresses these challenges through its multifactorial mechanisms. The technique involves foam dressing application within wound cavities, sealed with semipermeable membranes to create a contained microenvironment. Connection to a vacuum system establishes continuous negative pressure (typically − 125 mmHg), facilitating exudate removal and angiogenesis while inhibiting bacterial proliferation [23]. Since receiving FDA clearance in 1997, NPWT has demonstrated efficacy across diverse wound etiologies. Within maxillofacial surgery, its applications extend to complex reconstructions following necrotizing fasciitis (NF)-induced tissue defects. Notably, Chen et al. [11] applied it to the treatment of necrotizing fasciitis of the head and neck and achieved good results.

Previously, anatomical complexity and dynamic mobility of the craniocervical region posed technical challenges for NPWT dressing application and seal maintenance. However, in our experience, there were no difficulties in wound sealing by changing the application method of the semipermeable membrane. The secondary closure problems caused by beard growth and the exuberant secretion of sebaceous glands on the face can be solved by shaving the beard, cleaning the oil, and reapplying the semipermeable membrane material.

NPWT demonstrates several principal advantages over conventional tube drainage systems. Firstly, surgical trauma is significantly minimized. All patients in NPWT received bilateral submandibular transverse incisions (3–4 cm) permitting comprehensive spaces exploration. Traditionally, wide incisions in the LA have been used to obtain relief from the anaerobic environment within soft tissues, which means greater surgical trauma. In our treatment experience, this wide incision can be avoided, and adequate separation and exposure of the fascia are more important than debridement in the head and neck region. Secondly, drainage efficacy is enhanced. While catheter drainage suffices for localized abscesses, its effectiveness in the management of LA is debatable. There is no localized abscess cavity in the LA, and when incised, the infected spaces need to be adequately supported to prevent the cavity from collapsing, which can be achieved via NPWT. The inner surface of the involved cavity can be filled with a pruned NPWT sponge. Additionally, the traditional drainage method relies only on gravity, and the necrotic tissue and pus cannot be removed quickly, while the negative pressure principle of NPWT can eliminate the effect of gravity, remove these pathological exudates in time and keep the wound clean. Thirdly, the implementation of NPWT offers distinct advantages by eliminating the need for daily dressing changes. In conventional surgical wound management, routine dressing changes represent one of the most distressing experiences for patients, frequently associated with significant procedural discomfort. This innovative approach substantially reduces both the frequency of wound care interventions and the associated patient discomfort, while simultaneously decreasing the clinical workload through simplified wound management protocols. Among our patients, two underwent secondary debridement and NPWT device replacement. The remaining 16 patients required only a single session of surgical debridement combined with NPWT, demonstrating that the majority of cases achieved complete wound management through three sequential procedures during their hospitalization - a remarkably efficient treatment protocol given the severity of infections. The standardized intervention protocol consisted of: (1) Initial NPWT device implantation during primary debridement; (2) Secondary procedure for NPWT removal coupled with conversion to single-lumen negative pressure drainage; and (3) Final removal of the drainage system until achieving satisfactory wound closure. In terms of healthcare costs, although NPWT systems are costly, the reduced frequency of dressing changes, shortened wound healing time, and decreased length of ICU stay can offset this price increase. On the other hand, NPWT eliminates the unpredictability of traditional wound dressing changes, enabling the establishment of more standardized treatment protocols while also facilitating better cost control.

It is important to note that tracheotomy is considered a safe and acceptable invasive procedure for airway obstruction due to severe neck infection, but with the advancement of advanced endotracheal intubation techniques and airway management methods, tracheotomy is no longer advocated even for critically ill patients as long as the trachea can be removed [23–25]. Tracheotomy was not performed in any of our patients, and the swelling of the floor of the mouth and neck was quickly relieved after NPWT. Only one patient was retained for endotracheal intubation until 48 h after surgery, and the rest were controlled within 24 h. Nearly half of the patients were removed immediately after surgery, and no airway obstruction occurred. This may also be related to the negative pressure environment formed by NPWT, which rapidly reduces local tissue edema. These advantages need to be investigated in future clinical studies.

This study has several limitations. First, although NPWT demonstrates multiple advantages over conventional methods in LA, the small sample size in the control group may introduce statistical bias, necessitating validation through large-scale comparative studies. Second, the analysis of quantitative indicators (e.g., pain scores, scar quality, incision dimensions, and inflammation control) relies predominantly on descriptive presentation rather than rigorous statistical data processing, which weakens the objectivity of the conclusions.

In conclusion, NPWT demonstrates successful application in the treatment of LA, achieving superior clinical outcomes compared to conventional surgical approaches. This modality significantly reduces healing time, shortens ICU stays, minimizes surgical trauma through limited incisions, and alleviates procedural discomfort by decreasing dressing change frequency. Furthermore, NPWT implementation reduces clinician workload. Based on its advantages, NPWT should be considered as a viable approach in the management of LA. Future randomized controlled trials should confirm NPWT’s superiority in larger cohorts.

## Data Availability

All data supporting the findings of this study are available within the paper, with the exception of certain datasets containing protected health information that are not publicly available due to patient privacy considerations.
